# Reply to “Critical appraisal of “Proposal for the Hernia ASCEND Hugo™ RAS training pathway: acquisition of skills by comprehensive exercise‑based nimbleness and dexterity training””

**DOI:** 10.1007/s11701-025-02886-1

**Published:** 2025-10-10

**Authors:** Francesco Brucchi, Gianlorenzo Dionigi, Filip Muysoms

**Affiliations:** 1 University of Milan, Via Festa del Perdono 7, Milan, Italy; 2 Division of General and Endocrine Surgery, Istituto Auxologico Italiano, IRCCS (Istituto Di Ricovero E Cura a Carattere Scientifco), Milan, Italy; 3 Department of Pathophysiology and Transplantation, University of Milan, Milan, Italy; 4 Department of General Surgery, AZ Maria Middelares, Ghent, Belgium

**Keywords:** Robotic surgery, Hernia repair, Surgical education, Proficiency-based progression, Simulation, Hugo RAS

## Abstract

We appreciate the constructive comments by Haider et al. regarding our proposal for the Hernia ASCEND Hugo™ RAS training pathway. Their observations underscore key priorities for the ongoing refinement and validation of structured robotic hernia training. In our reply, we emphasize that the ASCEND framework is grounded in the Proficiency-Based Progression (PBP) model, which has demonstrated measurable improvements in surgical performance and safety across multiple specialties. We clarify that while our initial report did not include outcome data, multicenter validation studies linking ASCEND milestones to clinical endpoints are in progress. Moreover, we address concerns about generalizability and cost, reaffirming that ASCEND was independently designed by surgical educators and conceived as a modular and scalable program adaptable to diverse healthcare settings. We share the view that long-term validation should be guided by independent scientific societies to ensure neutrality and global comparability. Through these initiatives, ASCEND aims to contribute to the standardized, outcome-driven, and accessible adoption of robotic hernia surgery worldwide.

Dear Editor,

We thank Haider et al. [1] for their thoughtful critical appraisal of our article on the Hernia ASCEND Hugo™ RAS training pathway [2]. We greatly value their constructive feedback, which highlights important dimensions for the refinement and future validation of structured robotic hernia training.

## Clinical outcome validation

We fully agree that the ultimate validation of any training program must rest on patient outcomes. As emphasized in our article and in our recent systematic review [3], existing data remain fragmented, with few studies directly linking curricula to operative outcomes. The ASCEND pathway, however, is founded on the Proficiency-Based Progression (PBP) model [4, 5], in which errors relevant to robotic hernia surgery (e.g., mesh malposition, vascular injury, or nerve entrapment) are explicitly defined and systematically avoided before progression. PBP has consistently demonstrated reduced errors and improved performance across surgical domains [6, 7]. While our article did not aim to present outcome data, multicenter validation of ASCEND milestones against clinical endpoints is already underway and will remain a key priority.

In this context, our recent prospective comparison between the Hugo™ RAS and the da Vinci Xi systems for robotic inguinal hernia repair (r-TAPP) showed no measurable learning curve in terms of operative time for a surgeon experienced with the da Vinci platform, while maintaining safety and demonstrating significant postoperative quality-of-life improvements [8]. These findings support the translational applicability of structured, proficiency-based training and reinforce the rationale of the ASCEND framework for safe and efficient platform adoption.

## Generalizability and independence

We acknowledge the importance of independent and internationally benchmarked standards. Although ASCEND was initially developed in collaboration with academic centers and with technical input from industry, its structure and educational milestones were independently defined by surgical educators. Our view is that credentialing and quality assurance should ultimately be stewarded by scientific societies such as the European Hernia Society, ensuring neutrality and global comparability. To this end, discussions with international collaborators are ongoing to promote broad benchmarking and harmonization across different healthcare systems.

## Cost, infrastructure, and accessibility

We recognize that access to high-fidelity simulators, porcine models, and robotic platforms entails substantial investment, which may limit availability in low- and middle-income countries. This concern is valid and aligns with our own reflections on scalability. Importantly, the ASCEND pathway was designed to be modular, so that low-cost metric-based simulations, virtual/augmented reality, and remote-learning alternatives can be incorporated when resources are constrained. Several such initiatives are currently being piloted to ensure equitable access and global feasibility.

In conclusion, we are grateful to Haider et al. for their constructive comments. Their observations reinforce the rationale of ASCEND as a reproducible framework while underscoring the need for rigorous outcome-driven validation, international benchmarking, and cost-sensitive adaptations. By addressing these priorities, the ASCEND pathway aspires to contribute to the safe, standardized, and globally accessible adoption of robotic hernia surgery.

## Data Availability

No datasets were generated or analyzed during the current study.
